# Supplementary material for: Fruit Pouch Consumption and Dietary Patterns Related to BMIz at 18 Months of Age
Source: Nutrients. 2021 Jun 30;13(7):2265. doi: 10.3390/nu13072265 (PMC8308466; doi:10.3390/nu13072265)
Supplement: Supplementary file 1 [file nutrients-13-02265-s001.zip › nutrients-1242679-supplementary.pdf]

Table S1. The NorthPop questionnaires response rates for early life dietary variables.

Variables	Response rate	
	%	n
**At 18 months**		
Fruit pouch[1]	69.6	1044
Fruit juice[1]	68.3	1024
SSBs[1,2]	68.3	1024
Whole fruit[1]	68.8	1031
MCD[1,3]	69.7	1045
Breastfeeding, duration	74	1110
**At nine months**		
MCD[1,3]	75.6	1133
Breastfeeding, yes/no	78.5	1177
**At four months**		
Breastfeeding, exclusive	79.6	1193

[1] One-month-recall food frequency questionnaire [2] SSBs = sugar-sweetened beverages. [3] MCD = milk cereal drink.
